# Complement factor B inhibitor LNP023 mediates the effect and mechanism of AMPK/mTOR on autophagy and oxidative stress in lupus nephritis

**DOI:** 10.1002/kjm2.12894

**Published:** 2024-10-12

**Authors:** Xi‐Mei Zhang, Ming‐Jie Qing, Xin‐Kuo Liu, Liang Peng

**Affiliations:** ^1^ Department of Nephrology The Second Affiliated Hospital of University of South China Hengyang China; ^2^ Department of Endocrinology The Second Affiliated Hospital of University of South China Hengyang China; ^3^ Department of Basic Medicine Yueyang Vocational and Technical College Yueyang China

**Keywords:** AMPK/mTOR, autophagy, LNP023, lupus nephritis, oxidative stress

## Abstract

This study investigated the impact of LNP023 on the AMPK/mTOR signaling pathway in lupus nephritis (LN) and its effects on autophagy and oxidative stress. A mouse model of LN was established, and renal injury was confirmed by assessing various LN markers, including antinuclear antibody, ds‐DNA, anti‐Sm antibody, and others. Mice were treated with LNP023, the AMPK activator AICAR, or the AMPK inhibitor dorsomorphin. Renal injury and fibrosis were evaluated using HE and Masson staining. Expression levels of AMPK, mTOR, LC3, Beclin1, and p62 were assessed by immunohistochemistry and Western blot. Oxidative stress and inflammatory markers were measured by polymerase chain reaction and enzyme‐linked immunosorbent assay. LN mice exhibited low AMPK/p‐AMPK and high mTOR/p‐mTOR levels, alongside significant renal injury, fibrosis, reduced autophagy, and elevated oxidative stress. LNP023 treatment improved these parameters, with enhanced effects when combined with AICAR. Conversely, dorsomorphin reversed LNP023's therapeutic benefits. The complement factor B inhibitor LNP023 promotes kidney health in LN mice by mediating the AMPK/mTOR pathway, promoting autophagy, and attenuating oxidative stress.

## 1 INTRODUCTION

Systemic lupus erythematosus (SLE) is an autoimmune disorder in which the immune system fails to recognize the body's own nuclear components, leading to widespread autoimmunity and damage to various tissues and organs.^1^ Among these, kidney injury is the most common, with lupus nephritis (LN) occurring in nearly 100% of SLE patients. The progressing to end‐stage renal disease varies depending on the specific type of pathology.^2^ LN primarily arises from inflammatory damage to renal structures, leading to renal insufficiency. This represents a severe organ manifestation in the context of SLE.^3^ Currently, LN treatment relies on hormonal and immunosuppressive biologics; however, this approach has a high likelihood of relapse, and the toxicity associated with immunosuppressive drugs remains a significant challenge.^4^ Therefore, understanding the underlying mechanisms of LN is essential, as it paves the way for the development of targeted therapies specifically for LN treatment.

The pathogenesis of LN is multifaceted, involving factors such as activation of the complement system in renal tissue, extensive deposition of immune complexes, and an overactive lymphocyte response.^5^ Recent research highlights the growing importance of autophagy and oxidative stress in the development of LN.^6^, ^7^, ^8^, ^9^, ^10^ Autophagy is a complex and tightly regulated cellular process that removes and recycles unwanted or malfunctioning cytoplasmic components by transporting them to the lysosome for breakdown.^11^ Under normal conditions, this cellular process plays an active role in protecting cells from damage by clearing out accumulated dysfunctional components.^12^ Autophagy is vital in nearly every aspect of the immune response, crucial for maintaining intracellular homeostasis and defending against external stresses.^13^ Conversely, oxidative stress results from excessive production of reactive oxygen species within cells, disrupting intracellular stability and triggering a cascade of cellular damage responses.^14^ Due to the immune system's loss of control over both innate and adaptive processes, LN leads to the attack of the body's own tissues and organs. Immune cells infiltrate renal tissues under the influence of chemokines, which can exacerbate inflammation and oxidative stress, resulting in severe kidney damage.^15^

The complement system, consisting of about 50 proteins, is crucial for identifying and eliminating self‐threats like apoptotic cells, initiating broad inflammatory responses, and sustaining innate immune defense.^16^ The complement system, which plays a key role in responding to tissue injury, is activated through three primary pathways: the classical, lectin, and alternative pathways.^17^ Complement factor B (CFB), a crucial component of the complement system, is implicated in the immunopathological processes of LN and SLE. It plays a vital role in activating the alternative complement pathway.^18^, ^19^ Although the significance of CFB in LN is well‐established, its specific role in regulating autophagy and oxidative stress during LN development remains unclear.

LNP023, a novel CFB inhibitor, is currently in clinical trials for various complement‐mediated diseases.^20^ This study aims to investigate the impact of LNP023 on the AMPK/mTOR signaling pathway in the renal tissues of LN patients, focusing on its regulatory effects on autophagy and oxidative stress. By thoroughly examining the role of LNP023 in LN, we aim to uncover potential mechanisms that modulate immunopathological processes and propose novel therapeutic approaches. Understanding how LNP023 influences the AMPK/mTOR signaling pathway will deepen our insights into its regulation of autophagy and oxidative stress, offering new targets and strategies for LN treatment. This study advances our understanding of LN pathogenesis and lays the groundwork for the development of new therapeutic drugs.

## 2 METHODS

### 2.1 Animal data

Thirty SPF female BALB/c mice at 8 weeks, were obtained from Nanjing University. The mice were acclimatized to a controlled environment with a temperature range of 20–26°C, 45%–50% humidity, and a 12‐h light/dark cycle, and with food provided ad libitum. The experiment was conducted in accordance with the principles of replacement, reduction, and refinement (3R) principle.

### 2.2 Animal model

All mice were acclimatized for 2 weeks before the experiment. They then received a single intraperitoneal injection of 0.5 mL of either pristane^21^, ^22^ or phosphate‐buffered saline (PBS). The mice were housed in a pathogen‐free environment and induced with pristane for 5 months. Successfully modeled mice were subsequently administered daily intraperitoneal injections with or without the CFB inhibitor LNP023 (20 or 30 or 40 mg/kg) or the AMPK activator AICAR, (100 mg/kg) or the AMPK inhibitor dorsomorphin (30 mg/kg) for an additional 2 months. Body weights were monitored, and serum samples were collected at Weeks 1, 3, and 5. After 7 months, the mice were humanely euthanized, and their kidneys were collected.

### 2.3 Animal grouping

After successful animal modeling, six mice injected with PBS, remaining unmodeled, were designated as the Control group. The modeled mice were then divided into several groups: the Model group (injected with pristane, *n* = 6); the LNP023 (−L; −M; −H) group (injected with pristane and LNP023 [20; 30; 40 mg/kg], *n* = 6); the LNP023 + AICAR group (injected with pristane and AICAR and LNP023 [30 mg/kg], *n* = 6); and the LNP023 + Dorsomorphin group (injected with pristane and Dorsomorphin and LNP023 [30 mg/kg], *n* = 6).

### 2.4 Biochemical detection

The concentrations of urea nitrogen (BUN), creatinine (CRE), 24 h proteinuria 5, serum complement C3, C4, cystatin C (CysC), and neutrophil gelatinase‐associated lipocalin (NGAL) were measured in mouse serum using a fully automated biochemical analyzer.

### 2.5 Enzyme‐linked immunosorbent assay

Kidney tissue homogenates were prepared, and the levels of superoxide dismutase (SOD), malondialdehyde (MDA), and glutathione peroxidase (GSH‐Px) were assessed by enzyme‐linked immunosorbent assay (ELISA) kits according to the manufacturer's instructions. Mouse serum was collected and the concentrations of Anti‐Sm antibody, antinuclear antibody (ANA) and anti‐double‐stranded DNA (dsDNA) antibody  were determined by ELISA kits according to the manufacturer's instructions.

### 2.6 Hematoxylin–eosin staining

Paraffin sections of kidney tissue were deparaffinized to water. They were stained using hematoxylin and eosin (HE), then dehydrated sequentially in xylene and ethanol, and finally observed under an ordinary light microscope.

### 2.7 Masson staining

Paraffin sections of kidney tissue were deparaffinized to water. Kidney tissue sections underwent a sequential staining process employing a Masson staining kit in accordance with the provided guidelines. Subsequently, the sections were exposed to 1% ice‐cold acetic acid for a duration of 1 min, followed by dehydration and sealing. Images of the stained tissues were captured utilizing digital confocal microscopy. The degree of fibrosis of renal tissue was analyzed using ImageJ software.

### 2.8 Immunohistochemistry

Kidney tissue, fixed in paraformaldehyde for 48 h, underwent a gradual dehydration process using a sucrose gradient before being embedded in OCT. The resulting 2‐μm frozen sections were washed with PBS and sealed in a solution of PBS with 10% Bovine Serum Albumin (BSA) for an hour. Subsequent to this, the sections were exposed to specified antibodies for a 2‐h incubation at 4°C, followed by 4',6‐diamidino‐2‐phenylindole (DAPI) staining, and then mounted onto slides. Imaging was carried out using a digital confocal microscope, and fluorescence intensity analysis was performed using ImageJ software.

### 2.9 Western blot

Radioimmunoprecipitation Assay (RIPA) lysis buffer, enhanced with 1 mg/mL protease inhibitor, facilitated protein extraction and quantification via a BCA kit. The proteins from each group were heat treated at 95°C for 10 min, separated on 10% Sodium Dodecyl Sulfate (SDS) gels, and transferred onto Polyvinylidene Fluoride (PVDF) membranes. These membranes were then blocked using 5% skimmed milk. Primary antibodies against microtubule‐associated‐proteinlight‐chain‐3 (LC3), Beclin1, Sequestosome 1 (p62), AMPK, mTOR, p‐AMPK, p‐mTOR, and GAPDH were applied at 4°C for 24 h, followed by a 2‐h incubation with anti‐rabbit/mouse Horseradish Peroxidase (HRP)‐coupled secondary antibodies at room temperature. Chemiluminescence signals were captured using Bio‐RadChemDoc with an enhanced chemiluminescence solution, and band intensity was assessed using ImageJ software.

### 2.10 Polymerase chain reaction

Tissues were processed to extract total RNA using the RNA Rapid Purification Kit, and subsequent reverse transcription was carried out using the PrimeScript RT kit as per the manufacturer's guidelines. Quantitative polymerase chain reaction (qPCR) assays utilized the SYBR Green PCR Master Mix. The relative abundance of the target mRNA was normalized to the endogenous control (β‐actin) using the 2^−ΔΔCT^ formula. The primer details for the experiments are presented in Table 1.

**TABLE 1:** Experiments.

Gene	Direction	Sequence (5′‐3′)
AMPK	F	ATGGACTTGGTGAGCGAGG
	R	TCATCCGAGTCCAGGTTGT
mTOR	F	GACACAGGGAGATCGGAAGG
	R	TGTAGGAGCTGGAAAGCCAA
β‐Actin	F	CATGTACGTTGCTATCCAGGC
	R	CTCCTTAATGTCACGCACGAT
IL‐6	F	AGAGGATACCACTCCCAACAGACC
	R	TGCCATTGCACAACTCTTTTCTCA
TNF‐α	F	CCTGTAGCCCACGTCGTAGC
	R	GGCAGCCTTGTCCCTTGAAGAGA
IL‐1β	F	GCAACTGTTCCTGAACTCAACT
	R	ATCTTTTGGGGTCCGTCAACT

Abbreviations: IL, interleukin; TNF‐α, tumor necrosis factor‐α.

### 2.11 Statistical analysis

GraphPad Prism 8.0 was employed for statistical analysis. The results, representing the mean ± SEM, were derived from three independent experiments, each conducted in triplicate. Statistical significance among groups was determined using one‐way Analysis of Variance (ANOVA), followed by Dunnett's test. A significance threshold of *p* < 0.05 was applied.

## 3 RESULTS

### 3.1 Establishment of a mouse model of nephropathy

**FIGURE 1:**
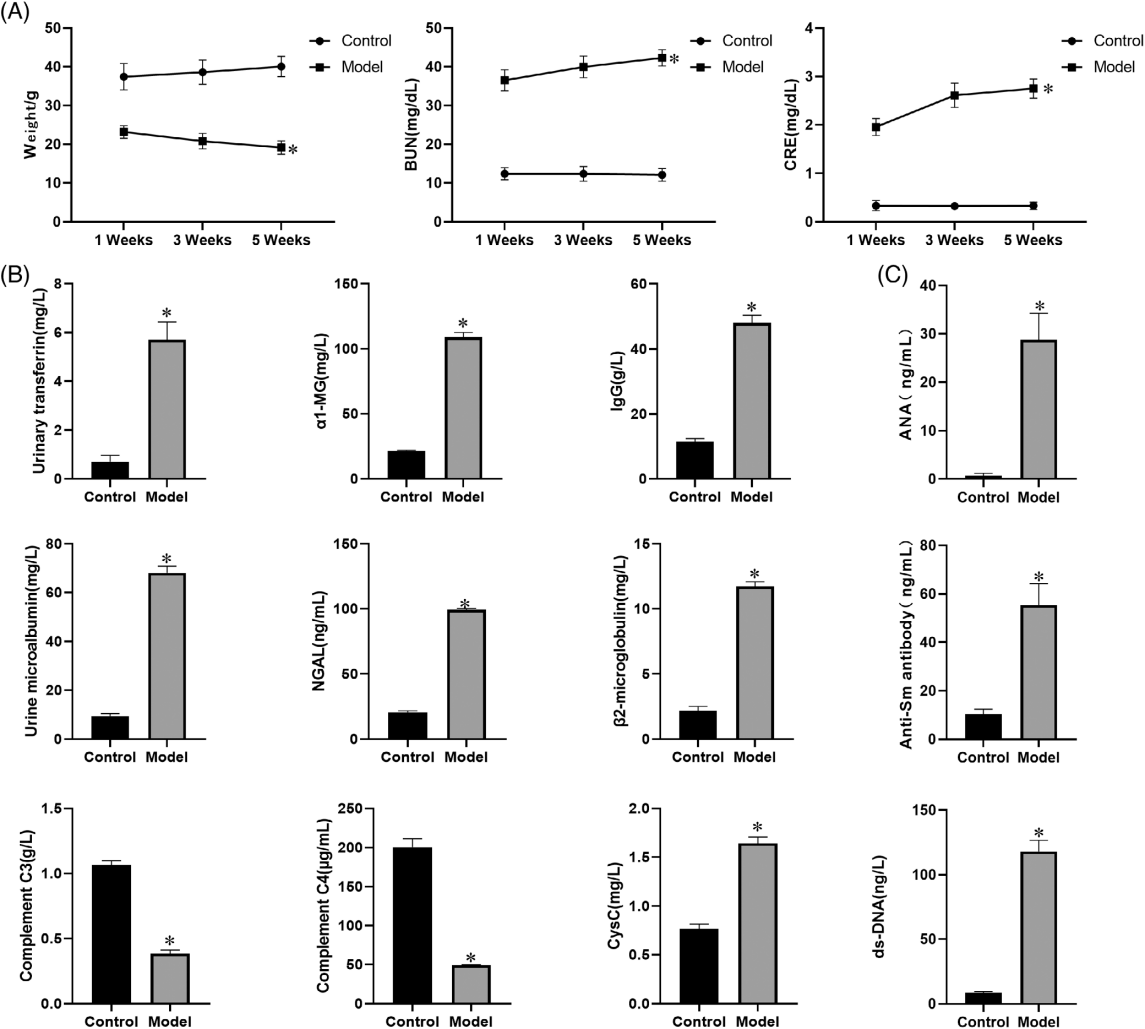
Establishment of a mouse model of nephropathy. (A) Mice were weighed at 1, 3, and 5 weeks and serum concentrations of urea nitrogen (BUN) and creatinine (CRE) were measured. (B) Detection of five items of 24 h proteinuria and serum complement‐related factor using a fully automated biochemical detector. (C) Detection of Anti‐Sm antibody, antinuclear antibody (ANA), and ds‐DNA concentrations in LN‐specific markers by enzyme‐linked immunosorbent assay. *p < 0.05 versus control (*n* = 6). CysC, cystatin C; NGAL, neutrophil gelatinase‐associated lipocalin.

Mice in the Model group exhibited skin rashes, erythema, ulcers, and vesicles on the face, ears, and back. Their hair became sparse, particularly behind the ears and on the back, where hair loss was severe, in contrast to the Control group. Body weight analysis revealed that mice in the Model group had significantly lower body weights compared with the Control group (*p* < 0.05). Additionally, serum analysis showed significantly elevated levels of urea nitrogen (BUN) and creatinine (CRE) in the Model group (Figure 1A) (*p* < 0.05). In the 24‐h proteinuria assessment, urinary transferrin, α1‐MG, Immunoglobulin G (IgG), β2‐microglobulin, and urinary microalbumin were also significantly higher in the Model group (*p* < 0.05). Serum analysis further indicated lower levels of complement C3 and C4, and higher levels of CysC and NGAL in the Model group compared with the Control group, with significant differences (Figure 1B) (*p* < 0.05). ELISA results showed that concentrations of Anti‐Sm antibody, ANA, and anti‐dsDNA antibody were significantly elevated in the Model group, confirming the successful establishment of the nephritis mouse model (Figure 1C) (p < 0.05).

### 3.2 Effect of LNP023 on renal injury and renal fibrosis in nephropathic mice

**FIGURE 2:**
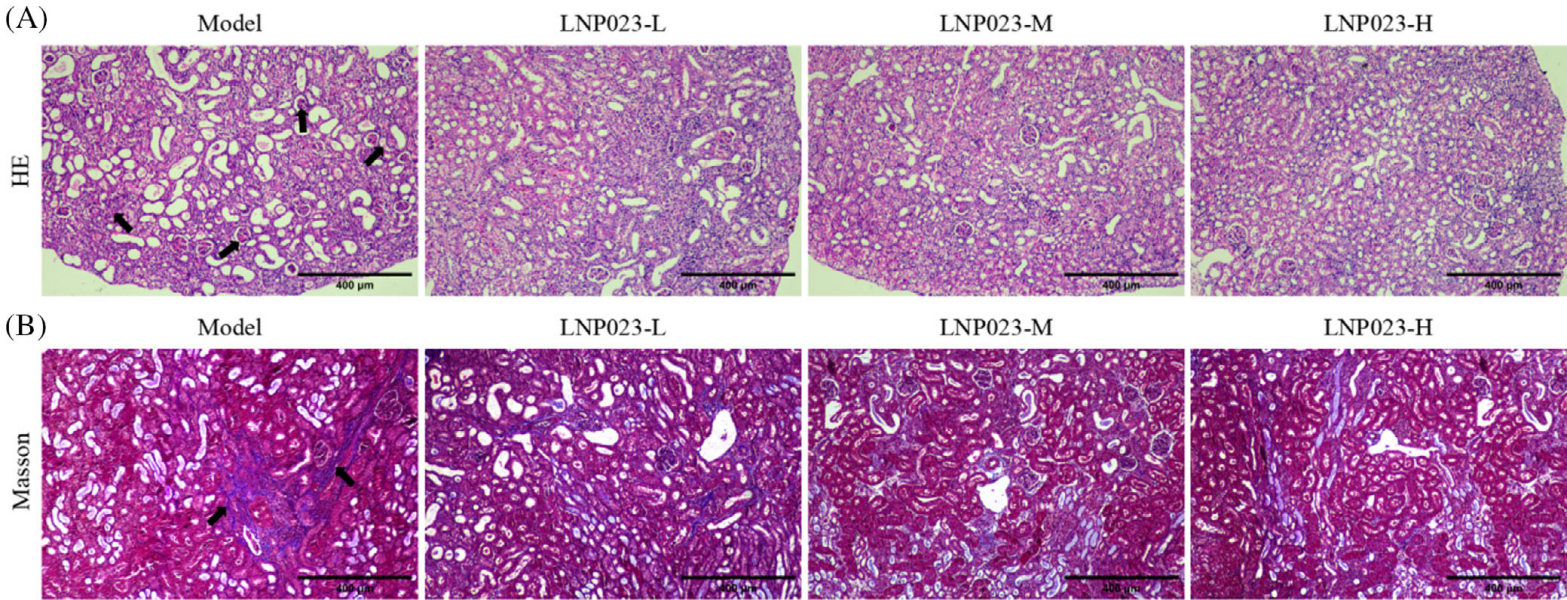
Effect of LNP023 on renal injury and renal fibrosis in nephropathic mice. (A) Hematoxylin and eosin (HE) staining to detect the extent of kidney tissue damage (100×, scale = 400 μm; “↑” shows areas of glomerular aggregation, tubular injury, inflammatory infiltration, and necrosis); (B) Masson staining to detect the degree of fibrosis in renal tissue (100×, scale = 400 μm; “↑” show collagen fibers, *n* = 6).

We assessed renal tissue damage and fibrosis in mice using HE and Masson staining. HE staining (Figure 2A) revealed significant damage in. the renal tissues of the Model group. The nuclei appeared purple‐blue, the cytoplasm was pink, and although the glomeruli and tubules were structurally intact, a large number of inflammatory cells were present. Renal interstitial blood vessels were dilated and congested, with extensive necrosis in the renal tubular epithelial cells and calcium salts deposits in the necrotic areas. After LNP023 treatment, renal tissue damage was significantly reduced in the LNP023 group, with the most pronounced improvement observed in the LNP023‐H group. Masson staining (Figure 2B) demonstrated a high degree of fibrosis in the renal tissue of the Model group, with collagen fiber hyperplasia was observed, and fibrous tissue proliferation was noted around blood vessels. Treatment with LNP023 significantly reduced the degree of renal fibrosis, with the greatest improvement seen in the LNP023‐H group. These results suggest that LNP023 effectively mitigates kidney injury and reduces renal fibrosis in nephritic mice.

### 3.3 Effects of LNP023 on AMPK/mTOR protein and autophagy in nephropathic mice

**FIGURE 3:**
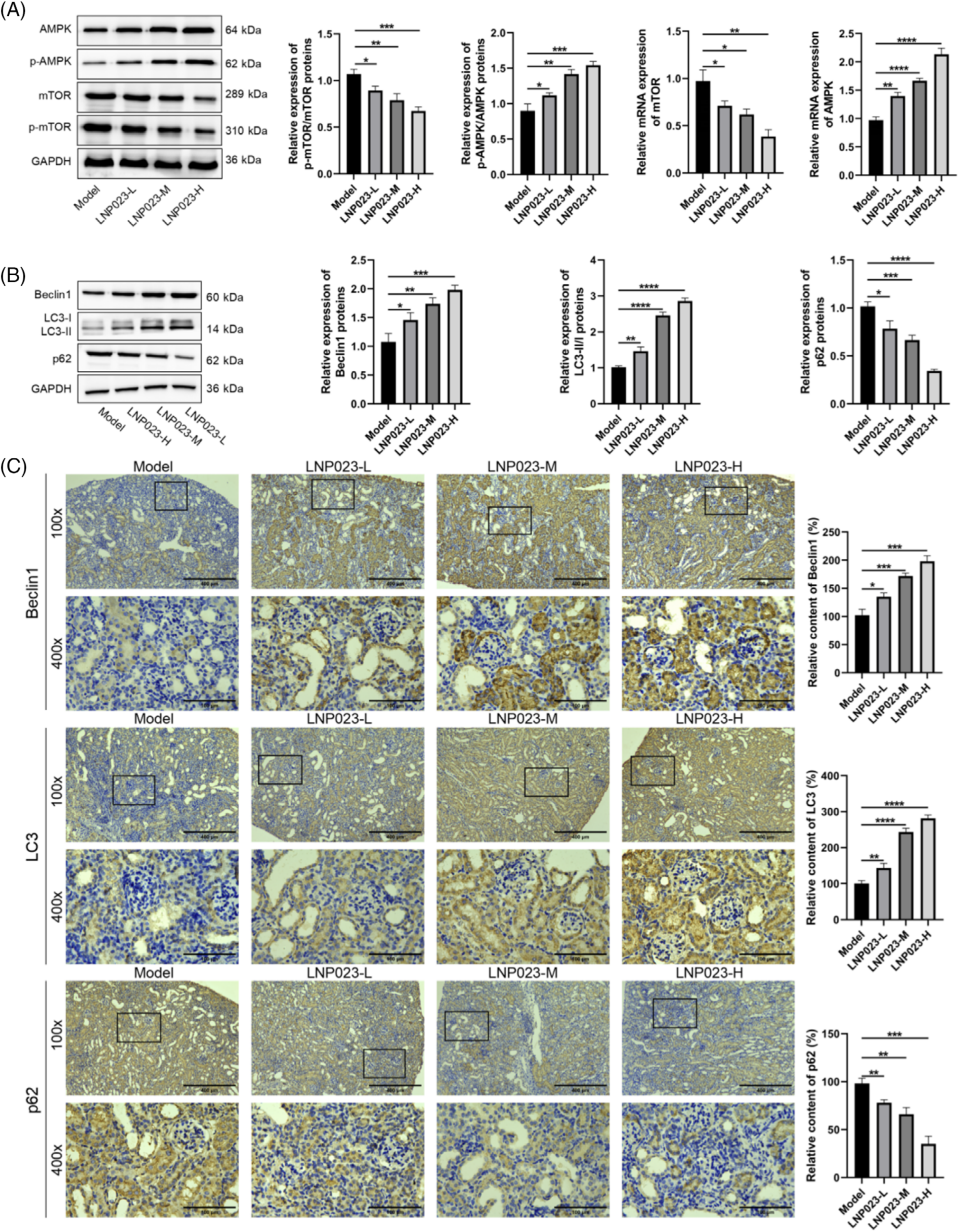
Effects of LNP023 on AMPK/mTOR protein and autophagy in nephropathic mice. (A) Western blot (WB) and polymerase chain reaction to detect the expression of pathway proteins (AMPK, p‐AMPK, mTOR, p‐mTOR) (using the relative expression levels of phosphorylation as the standard); (B) WB to detect the expression of autophagy‐related proteins (Beclin1, LC3, p62) (using the relative expression levels of GAPDH as the standard; LC3 was quantified using the LC3II/LC3I ratio as the standard); (C) Immunohistochemistry to detect the expression of autophagy‐related proteins (Beclin1, LC3, p62) in kidney tissues (100×, scale = 400 μm; 400×, scale = 100 μm). *p < 0.05 versus Model; **p < 0.01 versus Model; ***p < 0.001 versus Model; ****p < 0.0001 versus Model (*n* = 6).

To further explore the effects of LNP023 on the AMPK/mTOR pathway and autophagy in nephropathic mice, we analyzed the expression of the relevant proteins using PCR, Western blot (WB), and immunohistochemistry (Figure 3). PCR and WB assays showed that the LNP023 group significantly upregulated the expression of p‐AMPK/AMPK proteins and AMPK mRNA, while downregulated the expression of p‐mTOR/mTOR proteins and mTOR mRNA compared with the Model group (*p* < 0.05), with the most pronounced effect observed in the LNP023‐H group. These findings suggest that LNP023 modulates the AMPK/mTOR pathway. Furthermore, WB and immunohistochemistry assays revealed a significant increase in the expression of LC3 and Beclin1, along with a marked decrease in p62 expression, in the LNP023 group compared with the Model group (*p* < 0.05). Again, the LNP023‐H group exhibited the most significant changes. These results indicate that LNP023 enhances autophagy in the renal tissues of nephritic mice.

### 3.4 Effect of LNP023 on oxidative stress and inflammatory infiltration in nephropathic mice

**FIGURE 4:**
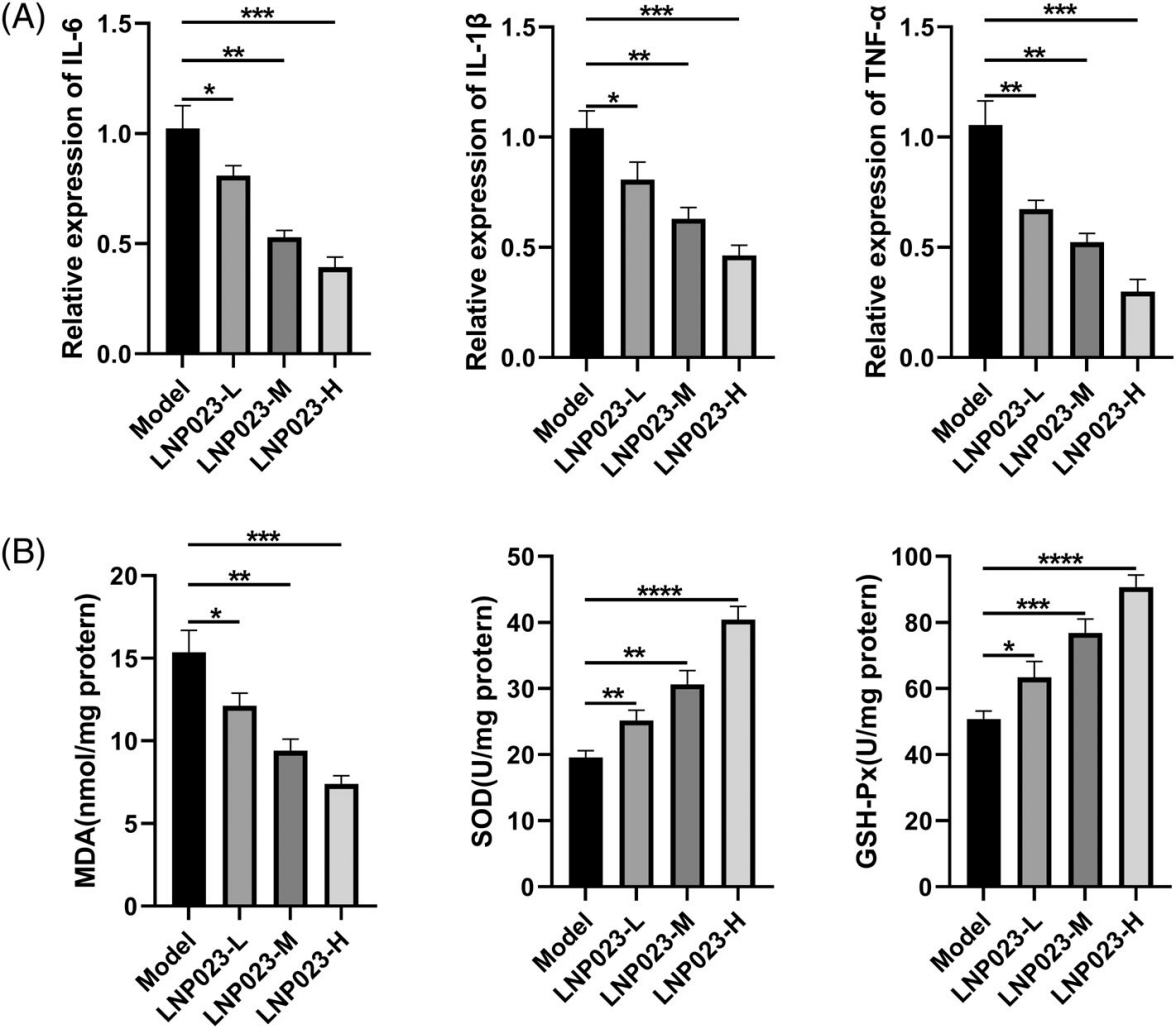
Effect of LNP023 on oxidative stress and inflammatory infiltration in nephropathic mice. (A) Polymerase chain reaction to detect the expression of inflammatory factors (interleukin (IL)‐6, IL‐1β, tumor necrosis factor‐α [TNF‐α]); (B) Enzyme‐linked immunosorbent assay to detect the content of oxidative stress‐related markers (malondialdehyde [MDA], superoxide dismutase [SOD], glutathione peroxidase [GSH‐Px]). *p < 0.05 versus Model; **p < 0.01 versus Model; ***p < 0.001 versus Model; ****p < 0.0001 versus Model (*n* = 6).

HE staining revealed extensive infiltration of inflammatory cells in the renal tissues of mice in the Model group. To assess inflammation and oxidative stress markers in renal tissues (Figure 4), we conducted PCR and ELISA assays. The results showed that mRNA expression levels of interleukin‐6 (IL‐6), IL‐1β, and tumor necrosis factor‐α (TNF‐α) were significantly lower in the LNP023 group compared with the Model group (*p* < 0.05). Additionally, the levels of MDA were markedly reduced, while SOD and GSH‐Px levels were significantly elevated (*p* < 0.05), with the most pronounced effects observed in the LNP023‐H group. These findings indicate that LNP023 effectively inhibits oxidative stress and inflammatory infiltration in the renal tissues of nephritic mice.

### 3.5 Intervention of the AMPK/mTOR pathway affects the effects of LNP023 on kidney injury and autophagy in lupus nephritis mice

**FIGURE 5:**
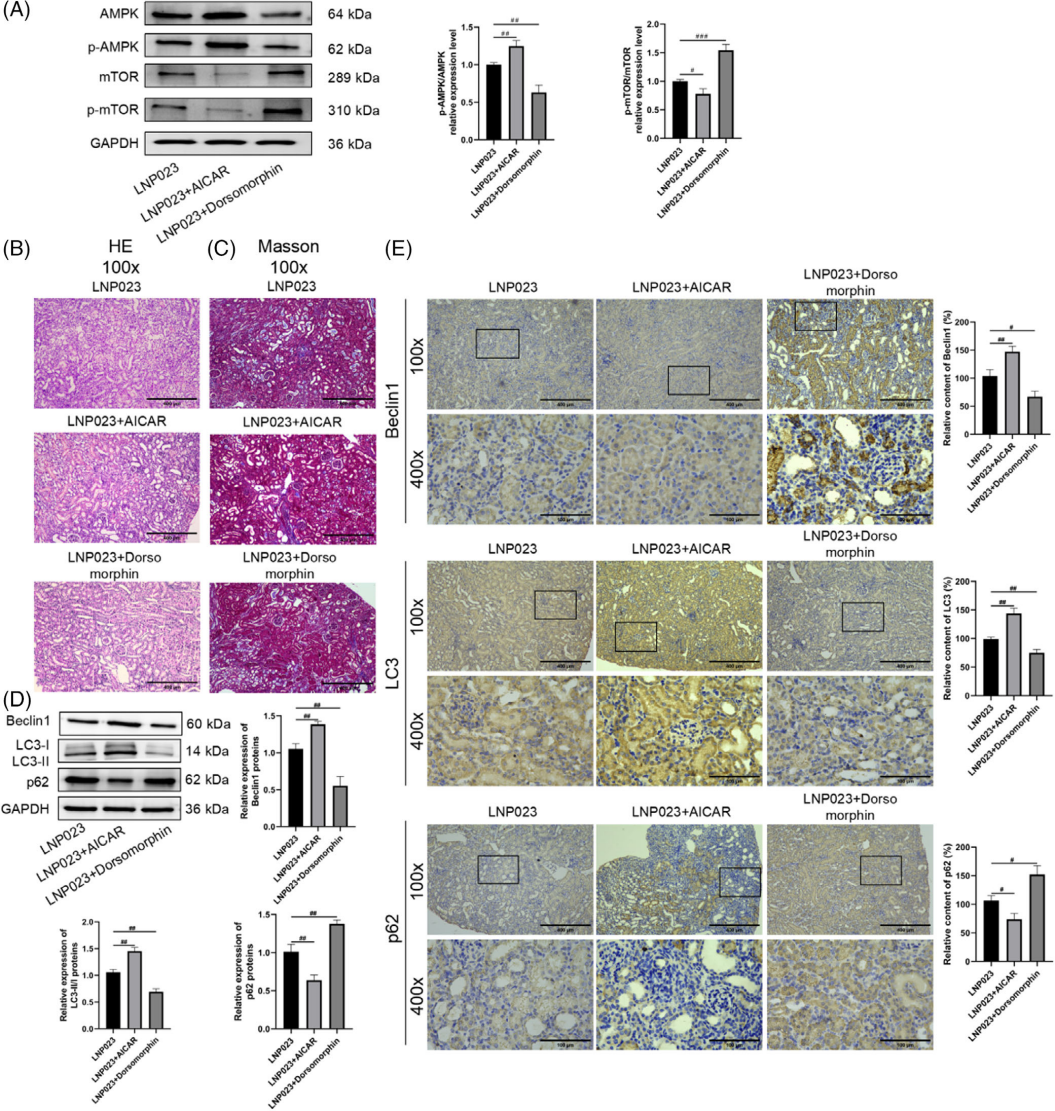
Intervention of the AMPK/mTOR pathway affects the effects of LNP023 on kidney injury and autophagy in lupus nephritis (LN) mice. (A) Western blot (WB) to detect the expression of pathway proteins (AMPK, p‐AMPK, mTOR, p‐mTOR; using the relative expression levels of phosphorylation as the standard); (B) Hematoxylin–eosin (HE) staining to detect the extent of kidney tissue damage (100×, scale = 400 μm); (C) Masson staining to detect the degree of fibrosis in renal tissue (100×, scale = 400 μm); (D) WB to detect the expression of autophagy‐related proteins (Beclin1, LC3, p62; using the relative expression levels of GAPDH as the standard; LC3 was quantified using the LC3II/LC3I ratio as the standard); (E) Immunohistochemistry to detect the expression of autophagy‐related proteins (Beclin1, LC3, p62) in kidney tissues (100×, scale = 400 μm; 400×, scale = 100 μm). ^#^ *p* < 0.05 versus Model; ^##^ *p* < 0.01 versus Model; ^###^ *p* < 0.001 versus Model (*n* = 6).

To investigate the specific mechanisms by which LNP023 affects renal injury and autophagy in renal tissue cells in nephropathic mice, we modulated the AMPK/mTOR pathway by administering AICAR (an AMPK activator) and Dorsomorphin (an AMPK inhibitor) to the LNP023‐treated group. The results (Figure 5) demonstrated that AICAR further promoted the expression and phosphorylation of AMPK, while Dorsomorphin reversed the LNP023‐induced upregulation of AMPK and p‐AMPK. Further research revealed that the LNP023 + AICAR group showed further improvement in renal injury and reduced renal fibrosis in nephropathic mice compared with the LNP023 group alone. Additionally, the levels of LC3 and Beclin1 in renal tissues were significantly increased, while the level of p62 was significantly decreased (*p* < 0.05). Conversely, in the LNP023 + Dorsomorphin group, the therapeutic effects of LNP023 were reversed, with a marked worsening of renal injury and increased renal fibrosis compared with the LNP023 group. In this group, the levels of LC3 and Beclin1 were significantly lower, and the level of p62 was significantly higher than those in the LNP023 group (*p* < 0.05). These findings suggest that LNP023 ameliorated renal injury and promoted cellular autophagy in renal tissues through the AMPK/mTOR pathway in nephropathic mice.

### 3.6 Intervention of the AMPK/mTOR pathway affects the effects of LNP023 on oxidative stress and inflammatory infiltration in lupus nephritis mice

**FIGURE 6:**
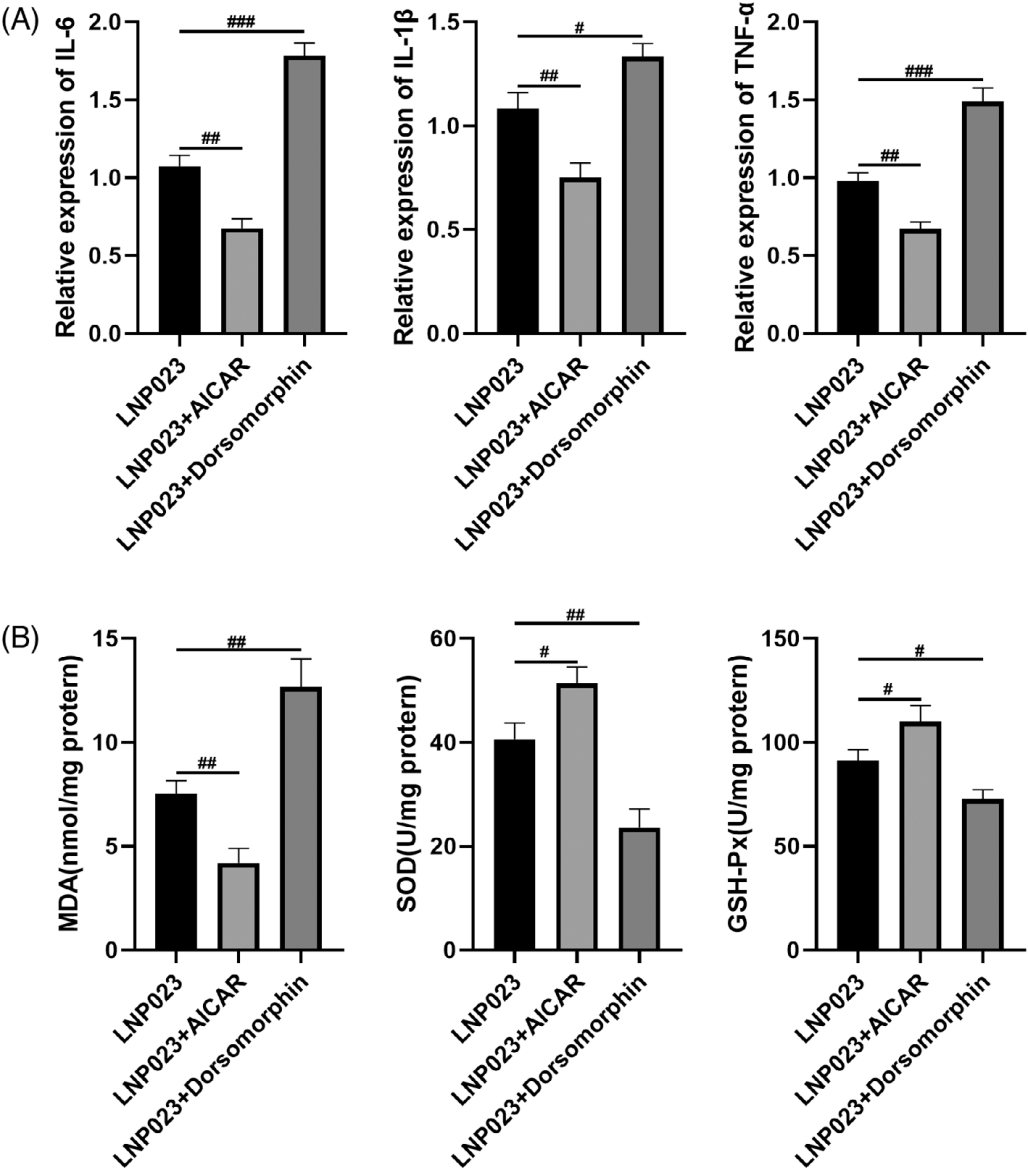
Intervention of the AMPK/mTOR pathway affects the effects of LNP023 on oxidative stress and inflammatory infiltration in lupus nephritis (LN) mice. (A) Polymerase chain reaction to detect the expression of inflammatory factors (interleukin (IL)‐6, IL‐1β, tumor necrosis factor‐α [TNF‐α]); (B) ELISA to detect the content of oxidative stress‐related markers (malondialdehyde [MDA], superoxide dismutase [SOD], glutathione peroxidase [GSH‐Px]). ^#^ *p* < 0.05 versus Model; ^##^ *p* < 0.01 versus Model; ^###^ *p* < 0.001 versus Model (*n* = 6).

Finally, we investigated the mechanisms underlying the effect of LNP023 on oxidative stress and inflammatory infiltration in LN mice. By modulating the AMPK/mTOR pathway, we observed (Figure 6) that the mRNA expression levels of IL‐6, IL‐1β, and TNF‐α were significantly higher in the LNP023 + AICAR group compared with the LNP023 group. Additionally, the level of MDA was significantly elevated, while the levels of SOD and GSH‐Px were significantly reduced (*p* < 0.05). In contrast, in the LNP023 + Dorsomorphin group, the therapeutic effects of LNP023 were reversed, with a significant decrease in the mRNA expression levels of IL‐6, IL‐1β, and TNF‐α in renal tissues compared with the LNP023 group. Furthermore, the level of MDA was significantly lower, while the levels of SOD and GSH‐Px were significantly higher compared with the LNP023 group (*p* < 0.05). These results indicated that LNP023 inhibited oxidative stress and inflammatory infiltration in renal tissues of LN mice through the AMPK/mTOR pathway.

## 4 DISCUSSION

SLE often presents with LN, a complication associated with significant morbidity and mortality.^1^ Effectively management of LN is essential to prevent progression to chronic kidney disease (CKD) and end‐stage renal disease (ESRD). Therefore, the short‐term therapeutic approach focuses on fully or partially reversing the clinical manifestations of renal injury. LN typically presents with mild to severe kidney inflammation caused by the accumulation of immune complexes containing autoantibodies. The current treatment strategy for LN involves induction and maintenance therapy. The induction phase lasts 3–6 months and uses glucocorticoids and potent immunosuppressive agents to reduce intrarenal inflammation and disrupt autoimmune pathways. The duration of the maintenance phase is not precisely defined.^23^ While this therapeutic strategy can slow the progression of LN to some extent, it is not entirely effective, with up to 30% of patients still advancing to ESRD. Additionally, the intensive use of these drugs may cause irreparable harm to the body.^4^ Research shows that complement activation is a key pathogenic mechanism in LN, with CFB playing a significant role as a crucial component of C3 and C5 convertases. LNP023, a potent CFB inhibitor, effectively suppresses the amplification of the alternative, classical, and lectin complement pathways. This inhibitor has demonstrated clinical utility in various complement‐mediated nephropathies.^24^

Autophagy, a programmed cellular process active in normal tissues, eliminates unwanted or damaged cellular components by transporting them to lysosomes for degradation and recycling. In SLE, studies have highlighted the critical role of autophagy, with significant implications for both innate and adaptive immunity.^25^ Under normal conditions, autophagy regulates antigen‐presenting cells, reduces the release of neutrophil extracellular traps, and suppresses the activation of effector T and B cells, thereby lowering autoantibody production and type 1 interferon signaling. However, in LN, autophagic activity is impaired, leading to increased production of inflammatory cytokines and reduced clearance of apoptotic cells, which contribute to the progression of LN. Additionally, autophagy is essential for maintaining homeostasis in renal tubular cells, and prolonged autophagy deficiency is a potential trigger for accelerated renal fibrosis.^12^

Autophagy is closely linked to the AMPK/mTOR pathway,^26^ which plays a key role in regulating autophagy across various diseases.^27^, ^28^ AMPK and mTOR are critical regulators of cellular metabolism, with most signaling occurring at the lysosomal membrane. AMPK promotes cellular metabolism, while mTOR inhibits it, and together, they orchestrate the autophagic process.

In this study, pristane‐induced mice exhibited notable reductions in body weight, elevated serum levels of BUN, CRE, CysC, and NGAL, along with decreased levels of complement C3 and C4. Additionally, there were increased urinary levels of transferrin, α1‐MG, IgG, β2‐microglobulin, and microalbumin, confirming the successful establishment of a nephritis mouse model. Subsequent analysis of AMPK, p‐AMPK, mTOR, and p‐mTOR expression in the Model group revealed decreased levels of AMPK and p‐AMPK, along with increase mTOR and p‐mTOR expression. However, LNP023 treatment reversed these effects, increasing AMPK and p‐AMPK expression while decreasing mTOR and p‐mTOR levels, indicating a regulatory effect on the AMPK/mTOR pathway. Co‐treatment with LNP023 and AICAR further elevated AMPK and p‐AMPK expression, suggesting a dual regulatory influence on the AMPK/mTOR pathway. Histological assessments, including HE and Masson staining, demonstrated significant renal injury and fibrosis in the Model group, which were ameliorated by LNP023 treatment, indicating its therapeutic potential. Analysis of autophagy‐related factors revealed reduced autophagic activity in the Model group, both of which were significantly alleviated by LNP023 treatment. Co‐treatment with AICAR further improved renal injury, fibrosis, oxidative stress, and inflammatory infiltration, while also enhancing autophagic activity. These findings suggest that the beneficial effects of LNP023 on nephritic tissues are mediated through the regulation of the AMPK/mTOR pathway.

In conclusion, these findings highlight the complex nature of LN, a multifaceted autoimmune disorder shaped by various factors. The AMPK/mTOR pathway emerges as a key player in LN pathogenesis, influencing critical cellular processes such as autophagy, oxidative stress, and inflammatory infiltration. The role of LNP023 in LN is significant and complicated, potentially influencing lymphocyte activity, complement system activation, immune complex deposition, and other mechanisms. Therefore, a comprehensive understanding of these interconnected factors is essential for fully grasping the intricacies of LN. The combined use of LNP023 and AICAR shows promise for effectively ameliorating LN.

## 5 CONCLUSION

The CFB inhibitor LNP023 promotes kidney health in LN mice by mediating the AMPK/mTOR pathway, promoting autophagy, and attenuating oxidative stress.

## CONFLICT OF INTEREST STATEMENT

The authors declare no conflicts of interest.
